# Phoenix: A Portable, Battery-Powered, and Environmentally Controlled Platform for Long-Distance Transportation of Live-Cell Cultures

**DOI:** 10.3389/fbioe.2020.00696

**Published:** 2020-06-26

**Authors:** Brittany N. Willbrand, Sylvia Loh, Caitlin E. O’Connell-Rodwell, Dan O’Connell, Devin M. Ridgley

**Affiliations:** SCORPIO-V Division, HNu Photonics LLC, Kahului, HI, United States

**Keywords:** cell therapy, live cell transport, CAR-T, mobile incubator, immunotherapy, SH-SY5Y cell line, stem cell

## Abstract

Despite the advent of advanced therapy medicinal products (ATMPs) in regenerative medicine, gene therapy, cell therapies, tissue engineering, and immunotherapy, the availability of treatment is limited to patients close to state-of-the-art facilities. The SCORPIO-V Division of HNu Photonics has developed the Phoenix-Live Cell Transport^TM^, a battery-operated mobile incubator designed to facilitate long-distance transportation of living cell cultures from GMP facilities to remote areas for increased patient accessibility to ATMPs. This work demonstrates that Phoenix^TM^ (patent pending) is a superior mechanism for transporting living cells compared to the standard method of shipping frozen cells on dry ice (−80°C) or in liquid nitrogen (−150°C), which are destructive to the biology as well as a time consuming process. Thus, Phoenix will address a significant market need within the burgeoning ATMP industry. SH-SY5Y neuroblastoma cells were cultured in a stationary Phoenix for up to 5 days to assess cell viability and proliferation. The results show there is no significant difference in cell proliferation (∼5X growth on day 5) or viability (>90% viability on all days) when cultured in Phoenix^TM^ and compared to a standard 5% CO_2_ incubator. Similarly, SH-SY5Y cells were evaluated following ground (1–3 days) and air (30 min) shipments to understand the impact of transit vibrations on the cell cultures. The results indicate that there is no significant difference in SH-SY5Y cell proliferation (∼2X growth on day 3) or viability (>90% viability for all samples) when the cells are subjected to the vibrations of ground and air transportation when compared to control samples in a standard, stationary 5% CO_2_ incubator. Furthermore, the temperature, pressure, humidity, and accelerometer sensors log data during culture shipment to ensure that the sensitive ATMPs are handled with the appropriate care during transportation. The Phoenix^TM^ technology innovation will significantly increase the accessibility, reproducibility, and quality-controlled transport of living ATMPs to benefit the widespread commercialization of ATMPs globally. These results demonstrate that Phoenix^TM^ can transport sensitive cell lines with the same care as traditional culture techniques in a stationary CO_2_ incubator with higher yield, less time and labor, and greater quality control than frozen samples.

## Introduction

Significant advances have been made to create advanced therapy medicinal products (ATMPs) in the form of regenerative medicine, cell therapies, gene therapies, tissue engineering, and immunotherapy. ATMPs are a growing treatment category with clinical applications for a variety of diseases and injuries, including cancer immunotherapy (Rosenberg and Restifo, 2015; Hosseinzadeh et al., 2018), brain and spinal cord injuries (Cox et al., 2017; Gabel et al., 2017), heart disease (Fan et al., 2019), pancreatitis (Ahmed et al., 2018), aneurysm (Adibi et al., 2016), paraquat poisoning (He et al., 2018), musculoskeletal disease (Law et al., 2019), erectile dysfunction (Reed-Maldonado and Lue, 2016), sickle cell disease management (Sadat-Ali et al., 2017), perianal fistulas in Crohn’s disease (Cheng et al., 2019), and diabetic foot ulcers (Lopes et al., 2018). Though initial animal and case studies appear promising, additional clinical trials are needed to fully characterize the efficacy and limitations of ATMPs in disease management including the standardization of treatment regimens between laboratory research and clinical applications (Borlongan, 2019; Brunet et al., 2019; Fukumitsu and Suzuki, 2019). Unfortunately, these treatment options require state-of-the-art laboratories to produce and maintain the necessary, highly sensitive cell and tissue cultures, dramatically limiting access to patients and researchers. Thus, facilities that offer cell therapy treatment would be inaccessible to patients with restrictive geographic accessibility, limiting market access (Mahalatchimy, 2011).

As cell therapies are becoming more mainstream within the medical community, there is a growing need to reliably transport these sensitive treatments to patients around the world. Currently, access to cell therapy technologies is limited for three primary reasons. (1) Due to the sensitive nature of these cell lines, the time and distance required for transport outside of a controlled environment will result in cell death and render the treatment ineffective. (2) Traditional incubators that are required to keep these cells alive within a R&D laboratory are neither portable, compact, nor energy efficient, rending this equipment ineffective for live-cell transport. (3) Finally, traditional cell transport requires the samples be cryogenically frozen to −150°C in liquid nitrogen (LN_2_) for preservation. LN_2_ cryopreservation during transport requires laborious, time-consuming biological manipulations prior to therapeutic use, which is neither efficient nor plausible to expect of on-site physicians and remote medical facilities.

Research in cryopreservation methods for bone marrow-derived mesenchymal stem cells (BM-MSCs) varied greatly from 50 to 100% viability depending on the cryopreservation method, cell passage number, and post-thawing testing time/method (Bahsoun et al., 2019). Moreover, these studies suggest that metabolic activity is altered and apoptosis is “evident” in the post-thaw BM-MSCs, though these pathways are not well-characterized (Bahsoun et al., 2019). For t-cells exposed to cryopreservation, recovery rates between 66 and 90% have been observed (Lemieux et al., 2016; Luo et al., 2017). These studies indicate that shipping cell cultures in LN_2_ increases the labor prior to and post-shipping as well as reducing the yield by up to 44 and 50% for t-cells and BM-MSCs, respectively. In addition, there is clear evidence to suggest that metabolic pathways are altered, and induction of apoptosis is increased for BM-MSCs. While more research is needed to fully understand the effects of cryopreservation on ATMP-like cells, these studies indicate that cryopreservation may have some negative impacts on cell function and performance. Thus, there needs to be a more efficient (cost and time) and cell culture friendly mechanism to transport ATMPs from production facilities to the patients in need.

Interestingly, there are a few examples of using simple CO_2_ incubators to transport reproductive cells from an R&D facility/location. In Asia, there are two examples of these simple incubators being used in veterinary applications to transport mink whale oocytes on a research vessel (Iwayama et al., 2005) and bovine embryos transported from Japan to China for nuclear transport (Dong et al., 2004). In both instances, CO_2_ gas was generated in a sealed and negatively pressured box via a chemical reaction with distilled water and effervescent granules. More recently, human pre-implantation embryos were transported in the LEC-960 portable incubator from Ukraine to Isreal (Levron et al., 2014). The LEC-960 was designed specifically for the transport of reproductive cells by using an aluminum heating block for multiple 0.5 mL vials. These studies demonstrate that it is possible to transport living cell cultures over long distances; however, these devices are designed to transport small quantities of reproductive cells to be used for assisted reproductive therapy (ART) applications. Unfortunately, there is no evidence that shows these simple portable incubators can transport sufficient volumes of ATMP-like cell cultures for R&D or therapeutic applications.

**FIGURE 1:**
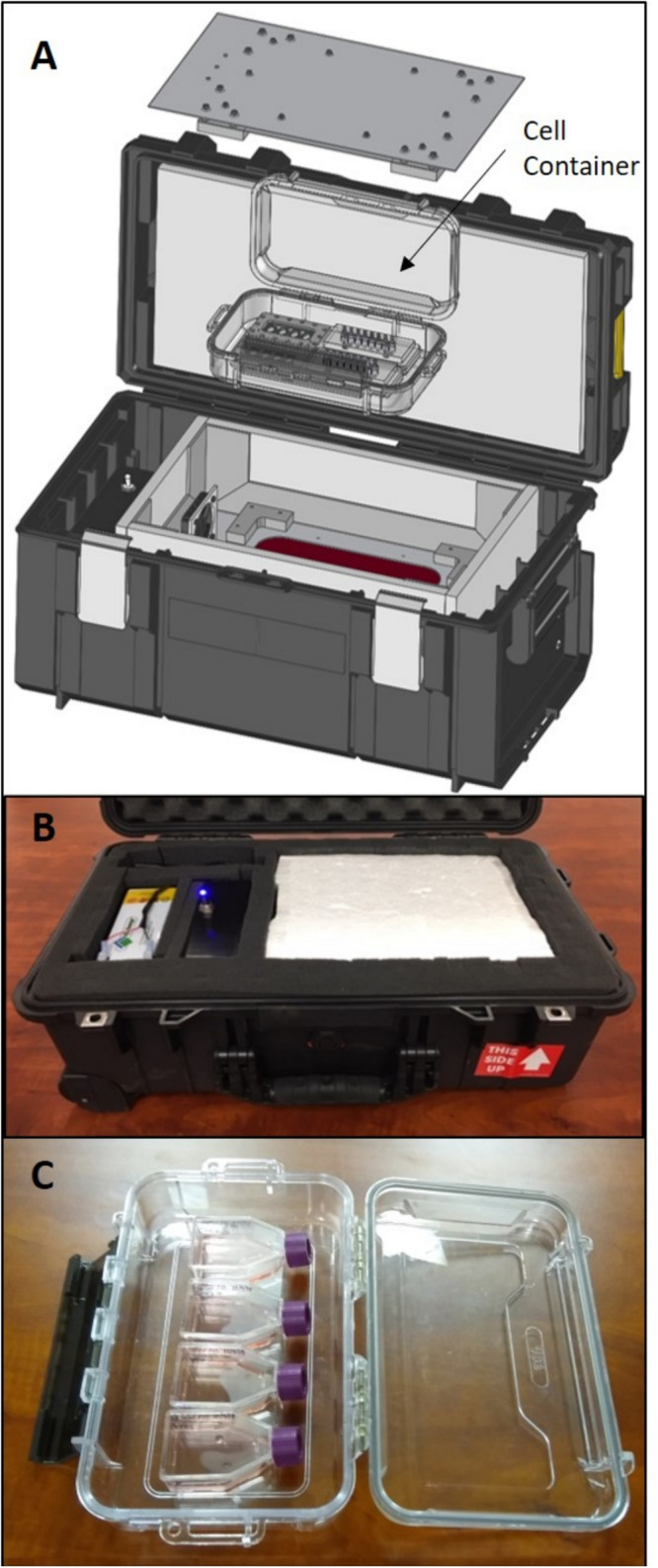
**(A)** A schematic illustration of the Phoenix system depicting the internal cell container that maintains a sterile environment for t-flasks (SCORPIO-V BioChips shown instead) and captures the 5% CO_2_ atmosphere. **(B)** The Phoenix system that was used for testing and to transport living neurons from Maui to West Texas for a test launch on Blue Origin’s New Shepard rocket. **(C)** The Phoenix cell container holding four t-12.5 flasks that were used for testing.

The SCORPIO-V team at HNu Photonics has developed a compact transport device that keeps cells healthy during shipments to facilitate live-cell transport of their cell lines for NASA-based research applications. Phoenix^TM^ is a live-cell portable incubator that is composed of the outer container that houses all electronics, power and heating elements, as well as the inner container (cell container) that is a disposable and sealed polymer box to capture the CO_2_ atmosphere and maintains the cell culture in a sterile environment (Figure 1). Previously, Phoenix^TM^ was developed and utilized to transport living neuroblastoma cells from Maui to the remote West Texas desert for a suborbital flight on Blue Origin’s New Shepard vehicle. Indeed, Phoenix technology is derived from the portable, compact, and automated thermal technology developed to perform life science investigations on the International Space Station. These technologies (BioChip SubOrbital Lab and Mobile SpaceLab) have successfully been deployed to Low Earth Orbit (LEO) for biological interrogations. While the Phoenix^TM^ has been used for space biology applications in the past, the technology has been harnessed and adapted to transport ATMP-like cell lines. The ability of Phoenix^TM^ to transport large volumes of ATMP-like cells (up to two t-75 flasks) with automated environmental control make it a viable option for the transport of cell therapies over existing portable incubators.

Phoenix^TM^ may revolutionize access of ATMPs to clinics and laboratories worldwide to create opportunities for accelerated research, new discoveries, and improved therapeutic accessibility. Although originally developed for space-based applications, Phoenix^TM^ meets a burgeoning market demand by providing a reliable and quality-controlled platform to transport cell therapies over long distances. This study investigates the efficacy of Phoenix^TM^ to maintain a healthy neuron-like cell cultures during several days of incubation and multiple transportation modes. Here, SH-SY5Y neuroblastoma cells were used as a proxy ATMP in order to examine the effects of long-term incubation and transport vibrations on cell proliferation, viability, and morphology. Furthermore, environmental factors such as relative humidity, triaxial acceleration, percent CO_2_, and temperature were monitored during each test to quantify the reliability of Phoenix^TM^ to maintain living cell cultures when compared to a standard CO_2_ incubator.

## Materials and Equipment

### Cell Culture

SH-SY5Y cells (ATCC) were cultured in DMEM/F12 (Sigma) supplemented with 10% fetal bovine serum (Sigma) and 1% antibiotic-antimycotic (Sigma). Cells were stored in a humidified incubator at 37°C with 5% CO_2_ to buffer media solution pH. Cells were cultured in a vented t-12.5 flask (VWR).

### Phoenix^TM^ System

An expanded view of Phoenix^TM^ is illustrated in Figure 1. Briefly, Phoenix is composed of the outer container that houses all electronics, power and heating elements, as well as the inner cell container that is a disposable and sealed polymer box that captures the CO_2_ atmosphere and maintains the cell culture in a sterile environment. It is important to note that the Phoenix system does not contain a CO_2_ source to prevent shipping constraints for air travel. The Phoenix system is designed to be transported as a “checked bag” to accompany a traveler if desired.

### Cell Viability and Proliferation

A live-dead assay was used to quantify cell viability with calcein AM (Thermo Fisher Scientific) and ethidium homodimer (Thermo Fisher Scientific). The cells were incubated in 4 μM of calcein AM and ethidium homodimer at 37°C for 20 min prior to imaging. Under a microscope, the cells were imaged at 25°C with brief exposures to 450 and 532 nm lasers for excitation of live and dead cells, respectively. Cell proliferation was quantified with a Countess II Automated Cell Counter (Thermo Fisher Scientific).

## Methods

### Cell Culture

Cells were passaged when confluency reached 80% with less than 10 passages for the cell cultures used for viability experiments and less than twenty passages for proliferation experiments. For each experiment, t-12.5 cell culture flasks were seeded at a density of 4.0 × 10^5^ cells per t-flask (5 mL total volume). Please see the Phoenix User’s Manual to determine what standard disposable cell culture containers can be used within the Phoenix System. In general, it will be up to the user to determine if a sealed of vented container is appropriate for transport of their cell line. Similarly, medium volume within the container should be considered as low volumes may induce adverse fluid-shear conditions on the given cell line.

### Phoenix and Transportation

Phoenix^TM^ was pre-heated to 37°C at least 30 min prior to each experiment. The cell container was sterilized with 70% isopropyl alcohol then stored in an open position inside a standard incubator enriched with 5% CO_2_. Prior to loading, media within t-flasks for both incubator treatment groups were supplemented with 25 mM of HEPES buffer solution (VWR) to buffer solution pH without the use of CO_2_. Once seeded t-flasks were added, the cell container lid remained in an open position. After 30 min of incubation, the cell container lid was moved to a closed position while inside the standard incubator. The cell container was then transferred to Phoenix^TM^. Ground transportation occurred between Kahului and Haiku, Hawaii, United States, where Phoenix was loaded inside a motor vehicle then transported 13 miles twice per day. Air transportation occurred in Kahului, Hawaii, United States. Phoenix was loaded into a motor vehicle and driven 4.2 miles to Maui Flight Academy at the Kahului International Airport. After loading Phoenix^TM^ into the cargo hold of a Cirrus SR22, a ∼30-min test flight was performed that included two take-offs (2.1 g), two 45° bank turns (1.6 g), two 60° bank turns (2.0 g), nosedive (0 g), and two landings (0–2.5 g). Additional values can be found in Figure 8. All flying aerobatics were performed at an altitude of 1500 ft.

### Experimental Design and Statistical Analysis

SH-SY5Y cells cultured in Phoenix^TM^ were compared to SH-SY5Y cells cultured in a traditional CO_2_ incubator. Cell viability and proliferation were examined after stationary incubation for 1–5 days, 1–3 days of ground transportation, and viability was examined following ∼30 min of air transportation. Thus, 17 experiments were performed in this study (eight proliferation and nine viability assays). There were four replicates for each experiment, which was defined as a single t-flask. There were eight technical replicates for the live-dead assay, which was defined as a single imaging frame. There were three technical replicates for cell counting, which was defined as a single frame. Technical replicates were averaged within each biological replicate for analysis. The error bars in figures represent the means ± standard error values. Prior to statistical analysis, histograms were examined, and tests were used to determine whether assumptions of normality and homogeneity of variance were violated (Shapiro–Wilk test and Levene’s test, respectively). The statistical model was a student’s *t*-test with a significance threshold of *p* < 0.05.

## Results

### Phoenix Data Logging and Stationary Incubation

**FIGURE 2:**
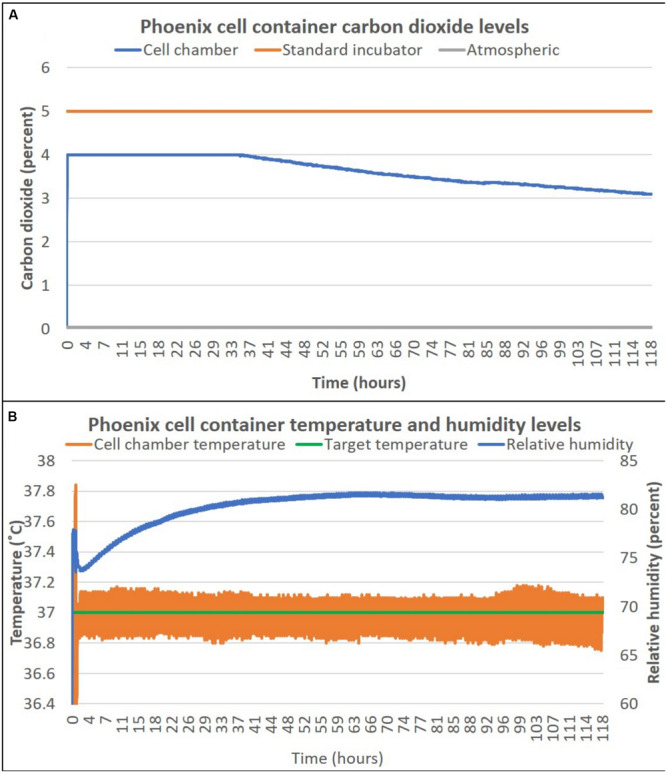
Phoenix^TM^ cell container carbon dioxide **(A)**, temperature and humidity **(B)** levels over 5 days. T-flasks were seeded with SH-SY5Y cells, incubated with media containing HEPES (25 mM), and loaded inside Phoenix^TM^ (*N* = 4) or a standard CO_2_ incubator (*N* = 4). Phoenix^TM^ was stored on a workbench with an exterior ambient temperature of ∼25°C and remained stationary for the entire 5-day period.

To assess the ability for Phoenix^TM^ to maintain the required environmental conditions to maintain a healthy cell culture, temperature, relative humidity, and percent CO_2_ were measured over 118 h (∼5 days) of live-cell culture. The cell container (as outlined in section “Methods”) was sealed in a traditional 5% CO_2_ incubator to capture the 5% CO_2_ atmosphere. This process is quick, but gentle to enable the capture of ∼5% CO_2_ for pH buffering of the cell culture during transport. The Phoenix CO_2_ sensor is only able to measure up to 4% CO_2_ due to sensor miniaturization requirements. Figure 2A shows that during the first 32 h of the experiment, the Phoenix CO_2_ sensor was saturated at 4% CO_2_. Thereafter, the percent CO_2_ decays at a rate of 0.012% CO_2_ per hour to reach 3% CO_2_ after 118 h (∼5 days). Temperature and humidity were also measured for 118 h (∼5 days). Prior to loading Phoenix^TM^ with a living cell culture, the device is turned on for 30 min to allow all components to reach a 37°C steady state. Thereafter, the cell container is loaded into Phoenix^TM^ and the live cell culture is maintained at 37 ± 0.25°C for the entire duration of the experiment (Figure 2B). Furthermore, the relative humidity within the cell container increases from 73 to 80% over the course of the 5-day experiment.

**FIGURE 3:**
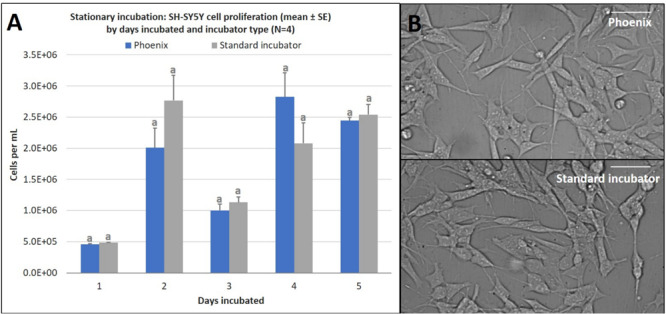
**(A)** SH-SY5Y cell proliferation (mean ± SE) by days incubated and incubator type following stationary incubation and **(B)** a representative 40x magnification image taken in brightfield after 5 days of incubation. The standard incubator was programmed to 37°C and 5% CO_2_ and Phoenix^TM^ was programmed to 37°C without CO_2_. Both incubators remained stationary throughout each experiment. Cell media was supplemented with 25 mM of HEPES in both treatment groups to buffer solution pH. Different letters indicate statistically significant differences within each experiment (Student’s *t*-test, *p* < 0.05). Scale bars in the upper right side depict 10 μM.

Once it was confirmed that Phoenix^TM^ could maintain the prerequisite conditions to maintain a living cell culture, SH-SY5Y cells were seeded into four T-12.5 flasks (*n* = 4) and incubated within Phoenix^TM^ for 1–5 days stationary and compared to replicate control samples maintained in a standard 5% CO_2_ incubator. There was no statistically significant difference in cell proliferation between Phoenix^TM^ and a standard 5% CO_2_ incubator for 1–5 days of incubation (Figure 3A). Assumptions of normality and homogeneity of variance were not violated, except for the 1-day dataset where the results of statistical analysis suggested that the sample was not derived from a population with a normal distribution (Shapiro–Wilk test, *p* = 0.0318). A *t*-test assuming equal variance was used for each analysis. On day 1, there were 4.6 × 10^5^ ± 1.4 × 10^4^ cells per mL in Phoenix^TM^ and 4.9 × 10^5^ ± 5.0 × 10^3^ cells in a standard 5% CO_2_ incubator; *t*(6) = 1.94, *p* = 0.1008. After 2 days of incubation, there were 2.0 × 10^6^ ± 3.1 × 10^5^ cells per mL in Phoenix^TM^ and 2.8 × 10^6^ ± 4.0 × 10^5^ cells in a standard 5% CO_2_ incubator; *t*(6) = 1.49, *p* = 0.1877. On day 3 of incubation, there were 1.0 × 10^6^ ± 1.1 × 10^5^ cells per mL in Phoenix and 1.1 × 10^6^ ± 8.4 × 10^5^ cells in a standard 5% CO_2_ incubator; *t*(6) = 0.98, *p* = 0.3660. After 4 days of incubation, there were 2.8 × 10^6^ ± 3.9 × 10^5^ cells per mL in Phoenix^TM^ and 2.1 × 10^6^ ± 3.3 × 10^5^ cells in a standard 5% CO_2_ incubator; *t*(6) = −1.47, *p* = 0.1922. On the final, fifth day of incubation, there were 2.4 × 10^6^ ± 5.3 × 10^4^ cells per mL in Phoenix^TM^ and 2.5 × 10^6^ ± 1.6 × 10^5^ cells in a standard incubator; *t*(6) = 0.56, *p* = 0.5966. Similarly, there were not any morphological anomalies or differences within the Phoenix^TM^ treatment or control (5% CO_2_ incubator) cell cultures (Figure 3B).

**FIGURE 4:**
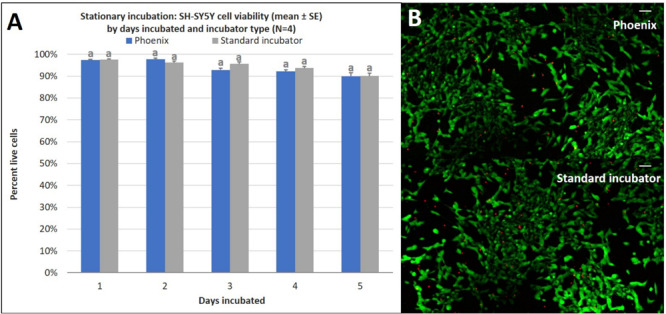
**(A)** SH-SY5Y cell viability (mean ± SE) by days incubated and incubator type following stationary incubation and **(B)** a representative live-dead overlay image taken at 10x magnification after 5 days of incubation. The standard incubator was programmed to 37°C and 5% CO_2_ and Phoenix^TM^ was programmed to 37°C without CO_2_. Both incubators remained stationary throughout each experiment. Cell media was supplemented with 25 mM of HEPES in both treatment groups to buffer solution pH. Different letters indicate statistically significant differences within each experiment (Student’s *t*-test, *p* < 0.05). A live-dead assay was used to quantify cell viability with calcein AM and ethidium homodimer with image post-processing to produce overlay. Scale bars in the upper right side depict 10 μM.

While Phoenix^TM^ incubation does not significantly alter cell proliferation, there may be differences in cell viability over extended durations. To assess this, SH-SY5Y cells were seeded into four T-12.5 flasks (*n* = 4) in the same manner as the cell proliferation experiment and treated with a Live-Dead assay on days 1–5 of stationary incubation within Phoenix^TM^ and compared to control samples (5% CO_2_ incubator). There was no statistically significant difference in cell viability between Phoenix^TM^ and a standard 5% CO_2_ incubator for 1–5 days of incubation (Figure 4). Since assumptions of normality and homogeneity of variance were not violated, a *t*-test assuming equal variance was used. On day 1 of incubation, there were 97 ± 1% viable cells in Phoenix^TM^ and 98 ± 1% viability in a standard 5% CO_2_ incubator; *t*(6) = 0.51, *p* = 0.6290. After 2 days of incubation, there were 98 ± 1% viable cells in Phoenix^TM^ and 96 ± 1% viability in a standard 5% CO_2_ incubator; *t*(6) = −1.67, *p* = 0.1450. On day 3 of incubation, there were 93 ± 1% viable cells in Phoenix^TM^ and 96 ± 1% viability in a standard 5% CO_2_ incubator; *t*(6) = 2.31, *p* = 0.0606. After 4 days of incubation, there were 92 ± 1% viable cells in Phoenix^TM^ and 94 ± 1% viability in a standard 5% CO_2_ incubator, *t*(6) = 1.43, *p* = 0.2039. On the final, fifth day of incubation, there were 90 ± 2% viable cells in Phoenix^TM^ and 90 ± 1% viability in a standard 5% CO_2_ incubator, *t*(6) = 0.07, *p* = 0.9489.

### Ground Transportation

**FIGURE 5:**
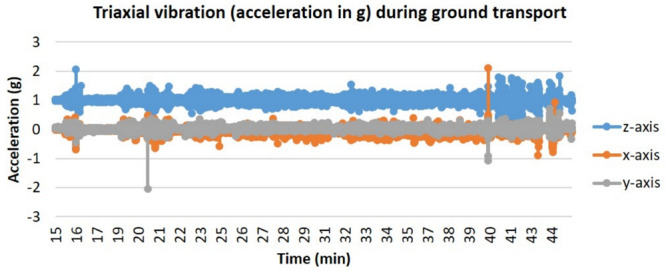
Triaxial vibration (acceleration in g) during ground transport. T-flasks were seeded with SH-SY5Y cells, incubated with media containing HEPES (25 mM), and loaded inside Phoenix^TM^ (*N* = 4) or a standard CO_2_ incubator (*N* = 4). Phoenix^TM^ was loaded into a motor vehicle and driven 13 miles between Kahului and Haiku, Hawaii, United States, with the depicted route driven twice per day of transit.

In addition to the providing the required temperature, humidity, and CO_2_ to a living cell culture, Phoenix^TM^ must also demonstrate that the vibrational loads on the device during transportation do not significantly alter cell proliferation, viability, or morphology. Additional tests were performed to culture cells in a moving vehicle for 1, 2, and 3 days to quantify cell proliferation and cell viability with respect to the acceleration loads the vehicle exhibits on the Phoenix^TM^ device. Triaxial vibration was recorded with an accelerometer over 72 h of the ground transportation experiment with vibration events observed on the *z*-axis between 0 and 2 g with a nominal 1 g static acceleration load. Similarly, in the *x* and *y* direction there was a nominal 0 g load with the majority of all vibrations occurring between −0.5 and 0.5 g for both axes (Figure 5).

**FIGURE 6:**
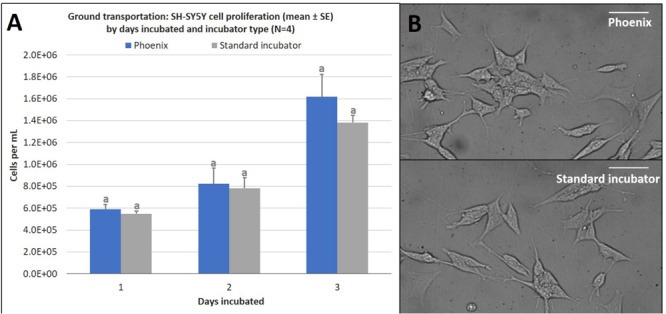
**(A)** SH-SY5Y cell proliferation (mean ± SE) by days incubated and incubator type following stationary incubation and **(B)** a representative 40x magnification image taken in brightfield after 2 days of incubation. The standard incubator was programmed to 37°C and 5% CO_2_ and remained stationary throughout the experiment. Phoenix^TM^ was programmed to 37°C without CO_2_ and was exposed to ground transportation. Ground transportation was performed on Maui, Hawaii, United States, with 13 mile segments twice per day. Cell media was supplemented with 25 mM of HEPES in both treatment groups to buffer solution pH. Different letters indicate statistically significant differences within each experiment (Student’s *t*-test, *p* < 0.05). Scale bars in the upper right side depict 10 μM.

Just as in the static cell culture tests, SH-SY5Y cells were cultured in four T-12.5 flasks (*n* = 4) and compared to replicate control samples in a standard 5% CO_2_ incubator. The results demonstrate that there is no statistically significant difference in cell proliferation for cells incubated in a standard 5% CO_2_ incubator when compared to cells exposed to ground transport within Phoenix^TM^ for 1–3 days (Figure 6). Since assumptions of normality and homogeneity of variance were not violated, a *t*-test assuming equal variance was used. After 1 day of incubation, there were 5.9 × 10^5^ ± 4.4 × 10^4^ cells per mL in Phoenix^TM^ and 5.5 × 10^5^ ± 2.3 × 10^4^ cells in a standard 5% CO_2_ incubator; *t*(6) = −0.84, *p* = 0.4308. After 2 days of incubation, there were 8.2 × 10^6^ ± 1.5 × 10^5^ cells per mL in Phoenix^TM^ and 7.8 × 10^6^ ± 9.6 × 10^4^ cells in a standard 5% CO_2_ incubator; *t*(6) = −0.23, *p* = 0.8285. After 3 days of incubation, there were 1.6 × 10^6^ ± 2.1 × 10^5^ cells per mL in Phoenix^TM^ and 1.4 × 10^6^ ± 6.7 × 10^4^ cells in a standard 5% CO_2_ incubator; *t*(6) = −1.09, *p* = 0.3163.

**FIGURE 7:**
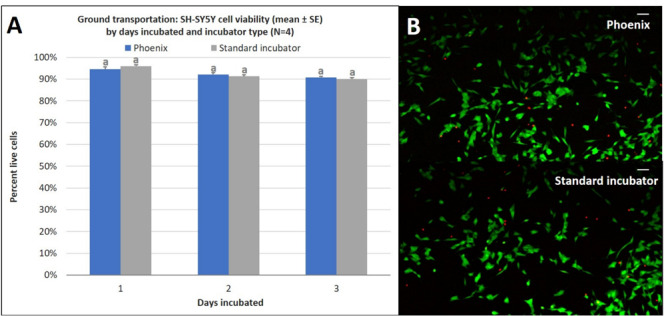
**(A)** SH-SY5Y cell viability (mean ± SE) by days incubated and incubator type following stationary incubation and **(B)** a representative live-dead overlay image taken at 10x magnification after 2 days of incubation. The standard incubator was programmed to 37°C and 5% CO_2_ and remained stationary throughout the experiment. Phoenix^TM^ was programmed to 37°C without CO_2_ and was exposed to ground transportation. Ground transportation was performed on Maui, Hawaii, United States with 13 mile segments twice per day. Cell media was supplemented with 25 mM of HEPES in both treatment groups to buffer solution pH. Different letters indicate statistically significant differences within each experiment (Student’s *t*-test, *p* < 0.05). A live-dead assay was used to quantify cell viability with calcein AM and ethidium homodimer with image post-processing to produce overlay. Scale bars in the upper right side depict 10 μM.

Similar to the ground cell proliferation results, there is no statistically significant difference in cell viability for cells incubated in a standard 5% CO_2_ incubator when compared to cells exposed to ground transport within Phoenix^TM^ for 1–3 days (Figure 7). Since assumptions of normality and homogeneity of variance were not violated, a *t*-test assuming equal variance was used. After 1 day of incubation, there were 95 ± 1% viable cells in Phoenix^TM^ and 96 ± 1% viability in a standard 5% CO_2_ incubator; *t*(6) = 1.22, *p* = 0.2677. On day 2 of incubation, there were 92 ± 1% viable cells in Phoenix^TM^ and 91 ± 1% viability in a standard 5% CO_2_ incubator; *t*(6) = −0.77, *p* = 0.4705. After 3 days of incubation, there were 0 ± 0% viable cells in Phoenix^TM^ and 0 ± 0% viability in a standard 5% CO_2_ incubator; *t*(6) = 0.00, *p* = 0.0000.

### Air Transportation

**FIGURE 8:**
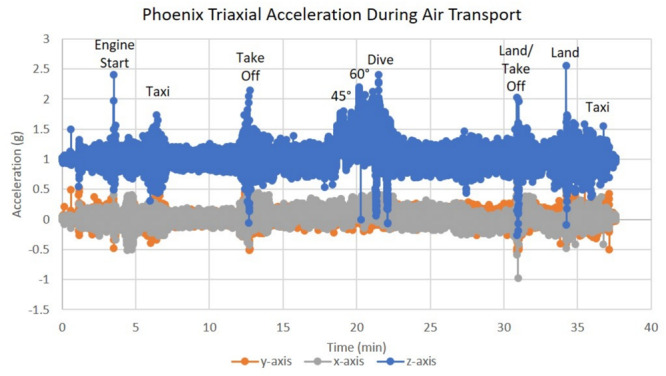
Triaxial vibration (acceleration in g) during air transportation. T-flasks were seeded with SH-SY5Y cells, incubated with media containing HEPES (25 mM), and loaded inside Phoenix (*N* = 4) or a standard CO_2_ incubator (*N* = 4). Phoenix^TM^ was loaded into a motor vehicle and driven 4.2 miles to Maui Flight Academy in Kahului, Hawaii, United States. After loading Phoenix into the cargo hold of a Cirrus SR22, a ∼30-min test flight was performed that included two take-offs, two 45° bank turns (1.41 g), two 60° bank turns (2.0 g), nosedive (0 –g), and two landings. All flying aerobatics were performed at an altitude of 1500 ft.

**FIGURE 9:**
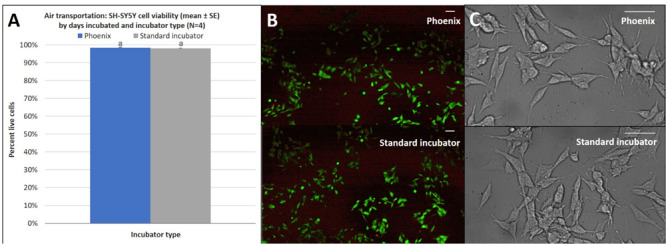
**(A)** SH-SY5Y cell viability (mean ± SE) by incubator type following air transportation, **(B)** a representative live-dead overlay image taken at 10x magnification, and **(C)** SH-SY5Y cell morphology observed at 40x magnification. The standard incubator was programmed to 37°C and 5% CO_2_ and remained stationary throughout the experiment. Phoenix^TM^ was programmed to 37°C without CO_2_ and was exposed to air transportation. T-flasks were seeded with SH-SY5Y cells, incubated with media containing HEPES (25 mM), and loaded inside Phoenix^TM^ (*N* = 4) or a standard CO_2_ incubator (*N* = 4). Phoenix^TM^ was loaded into a motor vehicle and driven 4.2 miles to Maui Flight Academy in Kahului, Hawaii, United States. After loading Phoenix^TM^ into the cargo hold of a Cirrus SR22, a ∼30-min test flight was performed that included two take-offs, two 45° bank turns (1.41 g), two 60° bank turns (2.0 g), nose dive (0 –g), and two landings. All flying aerobatics were performed at an altitude of 1500 ft. Different letters indicate statistically significant differences within each experiment (Student’s *t*-test, *p* < 0.05). A live-dead assay was used to quantify cell viability with calcein AM and ethidium homodimer with image post-processing to produce overlay. Scale bars in the upper right side depict 10 μM.

In addition to the acceleration loads of ground transport, Phoenix^TM^ will also experience the acceleration loads of air transportation. To assess how the SH-SY5Y cells will react to the acceleration environment of air travel, Phoenix^TM^ was flown on a Cirrus SR22 plane during a pilot training flight to assess the impact of high degree banking turns, nosedives, and multiple landing/takeoffs. Triaxial vibration was recorded with an accelerometer for the full duration of the test flight with peak vibration events observed on the *z*-axis at 0 and 2.5 g (Figure 8). Since assumptions of normality and homogeneity of variance were not violated, a *t*-test assuming equal variance was used. There was no statistically significant difference in SH-SY5Y viability for cells flown and incubated within Phoenix^TM^ when compared to a standard, stationary 5% CO_2_ incubator; *t*(6) = −0.96, *p* = 3747 (Figure 9A). After a ∼30-min flight, there were 98 ± 1% viable cells in Phoenix^TM^ and 98 ± 1% viability in a standard, stationary 5% CO_2_ incubator (Figure 9B). There were no observable differences in cell morphology for cells flown and incubated in Phoenix^TM^ when compared to a 5% CO_2_ incubator (Figure 9C).

## Discussion

The neuroblastoma cell line SH-SY5Y was used as a proxy ATMP in order to examine the effects of mobile incubation on cell proliferation, viability, and morphology. This study sought to verify the hardware capabilities of Phoenix^TM^ to maintain the required environmental conditions for healthy cell culture as well as examine SH-SY5Y viability and proliferation after stationary incubation in Phoenix^TM^ for 1–5 days. In addition, experiments were performed to examine SH-SY5Y viability and proliferation during ground and/or air transportation for up to 3 days. This work demonstrated that there were no significant differences in viability or proliferation observed between SH-SY5Y cells incubated in a standard CO_2_ incubator to cells transported via ground or air within Phoenix^TM^, suggesting that Phoenix^TM^ is an effective mobile incubator for live cell transport of ATMPs.

We show that the Phoenix^TM^ system can maintain the required environmental conditions under battery power to promote cell proliferation during both ground and air transport. The data sensors operate through a feedback loop and algorithm to maintain the required temperature (37°C) with minimal fluctuations (±0.5°C) to promote a healthy cell culture at near-physiological conditions. CO_2_ is traditionally used to buffer the medium pH and prevent pH shifts that naturally occur as a result of cell metabolism and growth, which can damage the cell culture over long times. In this instance, Phoenix^TM^ was able to capture the CO_2_ atmosphere from a standard 5% CO_2_ incubator and maintain the CO_2_ content over 3% during the 5-day experiment. While the Phoenix CO_2_ sensor can only read up to 4% CO_2_ due to spatial constraints, linear extrapolation of the percent CO_2_ indicates that the initial atmosphere at time 0 h was ∼4.4% CO_2_ with a decay rate of 0.012% CO_2_/h. When Phoenix^TM^ was operated in a similar mode to a standard 5% CO_2_ incubator (i.e., stationary), there were no significant differences in cell proliferation, viability, or morphology when compared to the control samples. Thus, the decrease in percent CO_2_ from ∼4.4 to 3% over 5 days did not have any discernable adverse effects on the SH-SY5Y cell culture when compared to the standard 5% CO_2_ atmosphere. Thus, from a pure incubator perspective, Phoenix^TM^ is just as effective as the traditional method of culturing mammalian cell lines in a biology laboratory for up to 5 days. It is important to mention, because there is some decrease of CO_2_ from day 0 to day 5, there may be some gas exchange (albeit minimal) through the polymer enclosure capturing the CO_2_ environment.

However, because Phoenix^TM^ is specifically designed to transport living cell cultures, it is important to investigate the effects of acceleration loads that the cells may experience during ground or air transportation. SH-SY5Y cells were specifically chosen as an ATMP proxy due to their required surface adherence and ability to be differentiated into a neuron-like cell culture. Thus, they would be more susceptible to vibrations or fluid shear stresses that may be present during transport than other non-adherent cell lines or therapies that may require Phoenix^TM^ for transport (t-cells, CAR-T, etc.). Nonetheless, the SH-SY5Y cells incubated in Phoenix^TM^ during transportation did not demonstrate any adverse effects when compared to the control samples. There were no significant differences in viability or proliferation between SH-SY5Y cells incubated in Phoenix^TM^ or a standard incubator regardless of incubation duration (1–5 days) or transportation method (stationary, ground, air). Cell viability remained above 90% for all experiments. Despite the 0 and 2.5 g encountered during air transport, there were no apparent differences in SH-SY5Y morphology observed for cells transported in Phoenix or cells that remained stationary in a standard incubator. Thus, the acceleration and vibration loads of travel do not appear to have detrimental effects on adherent cell lines within Phoenix^TM^ and would have less of an impact on suspension cell lines.

Phoenix^TM^ could alleviate one of the main hurdles of implementing wide-spread access to cell therapies: the freezing and thawing of biological samples, which reduces the quality of the product and requires laborious biological manipulations prior to and post-transport. Phoenix^TM^ will be a significant cost saving measure when ATMPs are deployed to clinics and patients globally by eliminating the time prior to freezing and the cell thawing/recovery period required to traditionally ship cell cultures (dry ice or LN_2_) by up to 3–14 days depending on the ATMP. Phoenix^TM^ can facilitate the implementation of parameters to quantify quality-controlled transport of live-cell therapies through data logging of environmental conditions with a multitude of embedded sensors. The need for a portable live-cell incubator is clear, but the exact implementation for all cell therapy applications requires further research. Indeed, multiple methodologies may be required to tailor the transport method of a given cell line/cell therapy product as not all cell lines behave the same. Future research should focus on characterizing additional cell lines such as mesenchymal stem cells and CAR-T cells for Phoenix^TM^ transport. The only way to ensure that this technology will achieve success is to gather input from subject matter experts and to characterize viability and proliferation for multiple ATMPs. Furthermore, additional research is needed to examine cell function at the point of use on a metabolic, rather than a morphological level, to ensure that treatment efficacy remains viable for transported cells.

Phoenix^TM^ live cell transport offers a time and cost saving alternative to traditional cell culturing and shipment techniques by providing a mechanism for rapid transportation with minimal biology preparation and exceptional data logging. The results of this study demonstrate that Phoenix^TM^ is an effective mobile incubator for live cell transport which could assist researchers, medical doctors, and patients with improved access to ATMPs. In addition, Phoenix^TM^ may enable long-distance and/or international collaborations to accelerate ATMP research and discovery within the research community. This research validates Phoenix^TM^ for live-cell transport of sensitive cell cultures with negligible effects from acceleration loads, atmospheric conditions, thermal maintenance, and culture pH shifts. Phoenix^TM^ introduces a new paradigm shift to the commercialization and implementation of widespread access to ATMPs.

## Data Availability Statement

The datasets generated for this study are included in the article/supplementary material.

## Author Contributions

DR conceived and supervised the study. DR and BW designed the experiments. BW carried out the experiments, performed the statistical analysis, and wrote the first draft of the manuscript. SL developed image analysis macros to quantify response variables. DR, DO’C, and CO’C-R critically revised the manuscript. All authors contributed to manuscript revision, read, and approved the submitted version.

## Conflict of Interest

BW, SL, CO’C-R, DO’C, and DR were employed by HNu Photonics LLC. The authors declare that this study received funding from HNu Photonics LLC. BW, SL, and DR were responsible for the study design, data collection, data analysis, and manuscript preparation. CO’C-R, DO’C, and DR were responsible for the final preparation of this manuscript and decision to submit for publication.
